# Percutaneous Nephrolithotomy under Ultrasound Guidance in Patients with Renal Calculi and Autosomal Dominant Polycystic Kidney Disease: A Report of 11 Cases

**DOI:** 10.1155/2017/3483172

**Published:** 2017-02-21

**Authors:** Xiao Wang, Xuecheng Yang, Xiulong Zhong, Zhenlin Wang, Senyao Xue, Weifeng Yu, Zhen Dong

**Affiliations:** ^1^Department of Urology, The Affiliated Hospital of Qingdao University, Qingdao, China; ^2^Department of Urology, Yidu Central Hospital of Weifang, Shandong, China

## Abstract

Nephrolithiasis accelerates the renal failure in the patients with ADPKD. In order to evaluate the role of percutaneous nephrolithotomy in management of calculus in these patients, 11 patients with autosomal dominant polycystic kidney disease and renal stones were included in the study. Two patients had bilateral renal stones. All patients were treated by percutaneous nephrolithotomy under ultrasound guidance. 13 percutaneous nephrolithotomy procedures were performed in 1 stage by the urology team under ultrasound guidance. 5 people received second operation with flexible nephroscopy in lateral position. The success rate and morbidity and mortality of the technique and hospital stay were recorded.* Results*. The puncture procedure was fully successful in all cases. The renal function improved in these patients. 5 patients had moderate fever after the surgery. 5 patients received flexible nephroscopy to take out the residual calculi. 2 persons had ESWL therapy after the surgery.* Conclusion*. PCNL is an ideal, safe, and effective method to remove the stones from those patients with no definite increase in the risk of complication. The outcome and stone-free rate are satisfactory comparable to the PCNL in the patients without ADPKD.

## 1. Introduction

Autosomal dominant polycystic kidney disease (ADPKD) is characterized by the progressive development of multiple renal cysts that destroy the renal parenchyma. It is the most common genetic disorder leading to end stage renal disease. This disease is caused by germline mutations in PKD1 (85%) and PKD2 (15%) and is typically diagnosed later in life than autosomal recessive polycystic kidney disease. Approximately 70% of patients with ADPKD will progress to renal failure at a median age of 56 [1]. In the early stages of ADPKD, most of the patients show a stable period of mild to moderate renal failure, which later on hastened by urinary infection and nephrolithiasis [2]. 28% patients with ADPKD may have nephrolithiasis in this period [3].

Although percutaneous nephrolithotomy is considered as the most effective treatment for the patients with larger upper urinary calculi in these days, there are still many people doubts about the safety and efficacy for this technique in the patients with ADPKD and nephrolithiasis. Some reports showed PCNL had the same therapeutic effect without increasing complication.

In order to show that PCNL under ultrasound guidance is an ideal, safe, and effective method to remove the stones from the patients with ADPKD and nephrolithiasis, we present our experience in the treatment of 11 patients with ADPKD and nephrolithiasis by PCNL under ultrasound guidance. The data of the success rate and intraoperative and immediate postoperative morbidity were assessed. We also evaluated the safety and efficacy of this procedure.

## 2. Materials and Methods

11 medical records of nephrolithiasis with ADPKD since 2010 were reviewed. Two patients had bilateral stones. Therefore, 13 PCNL procedures were performed under ultrasound guidance. All patients were evaluated with X-ray KUB (kidney, ureter, and bladder) and urinary ultrasonography. In view of the renal function, Urinary CT Scan and intravenous urogram were used for evaluating of the renal anatomy and size and location of the stone. The hemogram, urinalysis, urine culture and sensitivity, renal function test, liver function test, levels of serum electrolyte, blood glucose and lipids, and coagulation profile were performed. The patients with positive urinary culture received oral antibiotics therapy under sensitivity indication until urinary culture became sterile.

The PCNL were performed under general anesthesia in all patients. After anesthesia, the patients underwent ureteric catheterization under cystoscopy. The dilution of methylene blue was efflux freely into renal pelvis by the ureteric catheter. Then the patients were turned into prone position and received puncture procedure by the urologists under ultrasound guidance. Only the blue fluid refluxing from puncture needles was confirmed as correct puncture. The tract was dilated to 22 Fr by Amplatz fascial dilators. Rigid nephroscopy was used to complete the procedure. Pneumatic and ultrasound disintegration were performed in all patients.

The residual stones were evaluated with Urinary CT Scan 3 days after surgery. 5 people received second operation with flexible nephroscopy in lateral position. These patients got another Urinary CT Scan 3 days after the second surgery. The postoperation hemoglobin level and serum creatinine were assessed. The auxiliary procedure as ESWL was recorded. The successful treatment and postoperative complications were also recorded.

## 3. Result

The mean age of patients was 50 (range 32–68 years).  Table 1 showed the patients demographics and present symptoms. Of the 11 people, 8 were male and 3 were female. As to preoperative comorbidities, 6 patients had proteinuria; 5 patients had hypertension. 2 patients had no symptoms and were found incidentally. All the patients had normal renal function before operation. Mean serum creatinine was 1.01 mg (+0.18 mg).  Table 2 indicates the characteristics and location of the stone in these patients. 6 patients had large stone burden, 2 patients had partial staghorn stone, and 3 patients looked for surgery because of the ESWL treatment failure.  Table 3 showed the perioperative, operative, and postoperative characteristics and outcomes. The mean operative time is 105 min; 3 patients received transfusion for the correction of hematocrit. 9 cases got subcostal cutaneous puncture; 1 case received supracostal puncture; 2 cases required 2 cutaneous tract access both subcostal and supracostal in order to clean the stone as much as possible.  Table 4 showed postoperative characteristics and outcomes. One patient had heavy bleeding when the renal drainage was removed. The bleeding happened soon after the drainage tube was taken out; the same tube was inserted into renal pelvis through the previous path and the bleeding was controlled immediately. 5 patients had moderate fever after the surgery. Among these people, 3 persons had confirmed urinary infection by urinary culture. 5 patients received flexible nephroscopy to take out the residual calculi. 2 persons had ESWL therapy after the surgery. Even after these therapies, 8 patients had confirmed residual calculi by Urinary CT Scan. 3 persons had more than 5 mm residual calculi.

According to the Clavien-Dindo classification of surgical complications, 5 patients with fever after surgery are Grade I. 4 patients are Grade 2: one patient got paralytic ileus; 3 patients got urinary infection after the procedure. The patient with severe bleeding is Grade 3 who receives cystoscopy therapy without transfusion.

**Figure 1:**
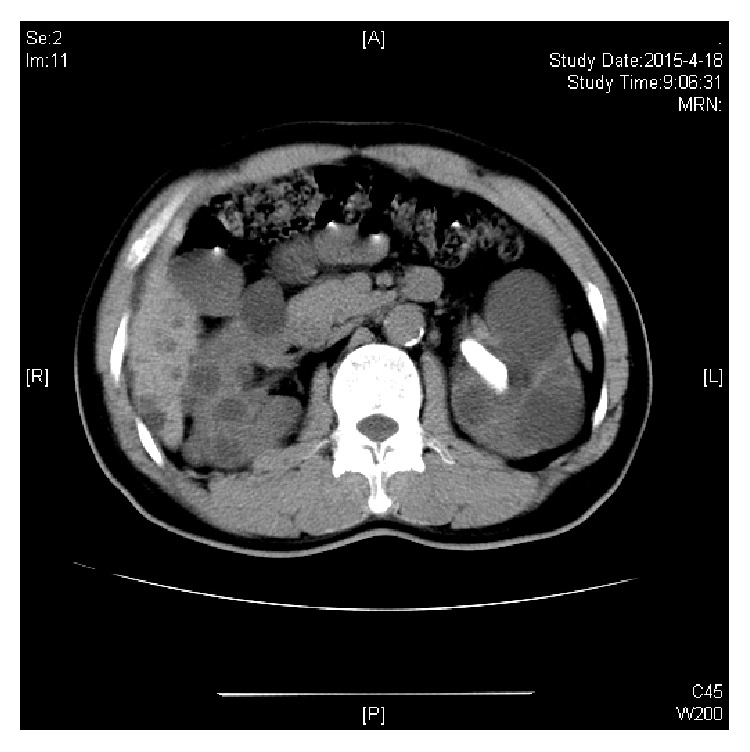
CT scan: left renal calculus with multiple cysts.

**Figure 2:**
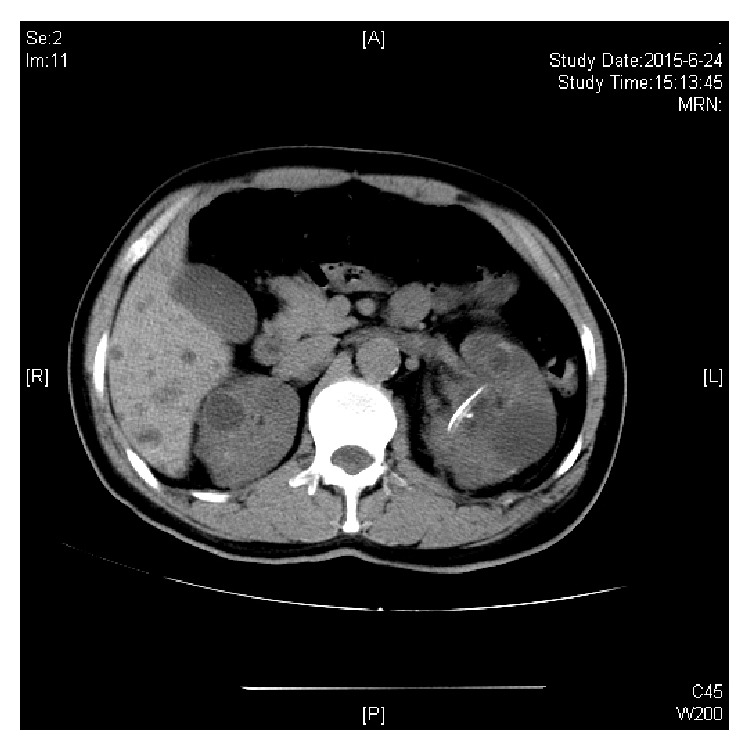
Nephrectomy tube after PCNL treatment.

Figure 1 showed a patient with left renal calculus and multiple cysts.  Figure 2 showed no calculi exist after PCNL treatment. He still kept the nephrectomy tube at that time.

## 4. Discussion

The incidence of renal calculi in patient with ADPKD is approximately 20 to 36%. Many ADPKD patients have urinary calculi. Nephrolithiasis aggravates the renal function damage and accelerates renal failure in these patients [4]. The common presenting symptoms of the calculi patients were hematuria and flank pain; 4 persons had hematuria. Among these patients, 2 persons had gross hematuria. Most patients also had proteinuria and hypertension, which should have cautious treatment before operation.

Percutaneous nephrolithotomy and ESWL are the main therapy for calculi patients with ADPKD. As ESWL treatment has some controversy with renal parenchymal damage [5, 6], nevertheless, the effects of ESWL were very poor in most reported research, in which the stone-free rate has been only 25–46% at 3 months. Multiple, large, branched calculus cannot be solved by ESWL therapy [7]. PCNL was considered to be the ideal method to remove the stones from the patients with ADPKD. We prefer ultrasound guidance which is popular nowadays. Under ultrasound guidance you can control the whole puncture pathway and avoid the influence of the cysts. As we all know, PCNL is a little difficult in these patients. The caliceal space can be elongated by the compressive effect of the parenchymal cysts. With the ultrasound guidance, multiple cysts influence the recognition of the caliceal aim. The dilution of methylene blue was very good method to confirm the puncture [8]. Only the continuous efflux blue liquid can be definite proof that you get the desirous calices. If you have not got the right calices, the use of ultrasound contrast agent may help you out of the dilemma.

In fact, it is a great challenge for us to establish the percutaneous tract under ultrasound guidance in these ADPKD patients. First, you need to confirm the calix you want to puncture and the cysts nearby under the ultrasound. In my opinion, the use of ultrasound contrast agent is not as good as they said in fact. When 20% meglumine diatrizoate was injected directly into the renal calices some turbulent flow reflection can be found by the ultrasound. Fluid in those polycysts never showed any turbulent flow reflection. Sometimes if the procedure cannot be achieved smoothly, you can puncture the stone directly. You may select the pathway through the cysts to the stone you aim at. The pathway may be long but there is not any bleeding danger but you should carefully fix you puncture tube in case of pathway loss.

The patients with recurrent kidney stone disease can greatly damage the glomerular filtration rate. The recurrence rates of the renal stone disease were as high as 50% within 5 years [9]. The use of flexible nephroscopy was very important in the PCNL with ADPKD in order to attain a complete stone-free status. As most patients with ADPKD have anatomical renal distortion and caliceal elongation, only one access tract cannot reach different portions of the collecting system in the PCNL procedure. Then comprehensive careful inspection of the renal collecting system should be performed with a flexible nephroscope [10]. Most portions of the collecting system can be reached by using a flexible nephroscope that might not have been reached with a rigid instrument. If bleeding makes the operation field not clear for the instant inspection, a second-look nephroscopy can be arranged.

The most concerned complication of PCNL performance is bleeding. Singh et al. reported that the average hemoglobin drop after the procedure was 2.1–3.3 mg/dL [2]. Most bleeding can stop automatically after the surgery, but some patients need to receive another performance of superselective angioembolization to control the bleeding. The incidence of acute bleeding requiring a blood transfusion is considered as the indication of safety for the patient with nephrolithiasis and AKPKD. Al-Kandari found the blood transfusion during or after the treatment was very low and there were no patient's needs for blood transfusion after their PCNL treatment in these 19 patients [11]. Thus the risk of nephrectomy is very rare and gross bleeding is not definitely associated with these complications in the present studies.

The renal function in these patients can be damaged by urinary obstruction and infection due to stones. Complete stone-free status is the best way to rescue renal function. The performance of PCNL had no definite influence in the renal function of these patients with AKPKD [12]. In this study, all the patients had normal renal function after operation. The mean serum creatinine level has no difference compared with that before operation in 3-month follow-up. The renal function keeps stable over the period of follow-up in this research. Srivastava et al. find the renal function of 22 patients with obstructive uropathy improved after PCNL performance, and there are no evidences of stone recurrence and renal function deterioration during a median follow-up of 43 months [7]. Paryani and Ather believe that serum creatinine level improved after the aggressive treatment, but the patients without any surgical intervention would have advanced to renal insufficient quickly [13]. Yet large multicenter studies are required to confirm this result in the patients with ADPKD and nephrolithiasis.

The patients with ADPKD and nephrolithiasis had great difficulty in achieving stone-free status. Umbreit et al. showed 82% patients were stone-free and 18% had small stone fragment remain. Nearly half patients received repeat percutaneous endoscopy in order to become stone-free [14]. Srivastava et al. believed relook PCNL and ESWL were needed to achieve stone-free status. Only 80% patients achieve stone-free after the first procedure. Nevertheless all patients were completely stone-free with relook and ESWL therapy [7]. Lei et al. claimed 69.9% patients were stone-free after the primary MPCNL. After the second-look MPCNL, only 4.3% of patients still had residual stone [15]. Compared with flexible ureteroscopy and holmium laser lithotripsy therapy, PCNL has the same stone-free rate. Liu et al. showed 84.6% stone-free rate after the first flexible ureteroscopy and holmium laser lithotripsy therapy. The stone-free rate can reach 92.3% after the second procedure [16].

Nishiura et al. found CT scan shows the most sensitivity and specificity compared with any other modalities in renal calculi evaluation [3]. The most components of the renal stone in the patients with ADPKD are calcium oxalate and uric acid. As many expanding renal cysts distort the intrarenal caliceal system, the urinary stasis and urinary crystals facilitated the formation and aggregation of renal calculus. The urinary oxalate and urinary crystallization were significantly higher in patients with ADPKD and nephrolithiasis. Lei et al. found the most common stone composition was calcium oxalate; uric acid and magnesium ammonium phosphate were also detected in some patients [15]. Liu et al. treat the patients with calculus and ADPKD with flexible ureteroscopy and holmium laser lithotripsy therapy, but the mean size of the stone was 5.6 mm [16]. However, flexible ureteroscopy and holmium laser lithotripsy therapy can be performed with the natural pathway which is better for the patients with renal function dysfunction and coagulation defect.

## 5. Conclusion

Nephrolithiasis accelerates the renal failure in the patients with ADPKD [4]. Based on the small number of cases and correspondence reports, PCNL under ultrasound guidance is an ideal, safe, and effective method to remove the stones from those patients with no definite increase in the risk of complication. The outcome and stone-free rate are satisfactory comparable to the PCNL in the patients without ADPKD.

## Figures and Tables

**Table 1:** Patient demographics and present symptoms.

Feature	Number	Pts (%)
Gender		
M	8	73
F	3	27
Age	32–68	
Average	50 ± 13	
Preop comorbidities		
Proteinuria	6	55
Hypertension	5	45
Presenting symptoms		
Hematuria	4	36
Flank pain	5	45
Asymptomatic	2	18
Stone burden		
Unilateral	9	82
Bilateral	2	18

**Table 2:** Indications for PCNL.

Indication	Renal units (*n*)
Large stone burden (>3 cm)	6
Partial staghorn stone	2
Large lower renal calculi (>2 cm)	2
Failed ESWL	3
Impacted stone at UPJ or lumbar ureter	0

**Table 3:** Stone characteristics and puncture locations.

Characteristics	Renal units
Units	
Side	
Right	6
Left	7
Stone location	
Renal pelvis	4
Caliceal	2
Multiple sites	7
Stone multiplicity	
Single	2
Multiple	9
Partial staghorn	2
Stone opacity	
Opaque	11
Lucent	2
Nature of stones	
Primary	13
Recurrent	0
Cutaneous tract access	
Subcostal	9
Supracostal	1
Both	2
Failed	0

**Table 4:** Postoperative characteristics and outcomes.

Characteristics and outcomes	
Severe hematuria	1
Fever	5
Paralytic ileus	1
ESWL	2
Flexible nephroscopy	5
Urinary infection	3
Effective residual calculi	3
Residual calculi	8
Preoperation Scr (mg/dL)	1.01 ± 0.18
Postoperation Scr (mg/dL)	1.08 ± 0.21
Preoperation Hb (mg/dL)	14.32 ± 2.12
Postoperation Hb (mg/dL)	12.86 ± 1.49
